# Emerging biomaterials and bio-nano interfaces in pulmonary hypertension therapy: transformative strategies for personalized treatment

**DOI:** 10.3389/fbioe.2025.1567783

**Published:** 2025-05-09

**Authors:** Xiaofa Chen, Lina Xu, Haiyan Shi

**Affiliations:** Department of Respiratory medicine, Nantong Third People’s Hospital, Nantong, China

**Keywords:** pulmonary hypertension, biomaterials, nanotechnology, drug delivery, tissue engineering

## Abstract

Pulmonary hypertension (PH) is still an aggressive and progressive illness with vascular remodeling and right heart failure despite the therapeutic advances made in the recent past. Biomaterials offer an attractive route to break the current therapeutic paradigms by inducing vascular repair, facilitating drug targeting, and allowing dynamic regeneration of tissue. This review taking an integrated approach investigates the revolutionary role played by novel biomaterials and bio–nano interfaces in PH treatment. We classify and evaluate several classes of biomaterial platforms including natural polymers, scaffolds based on synthetic polymers, extracellular vesicles (EVs), and stimulus-responsive systems with an emphasis on both underlying mechanisms and clinical relevance. We further address the progress made in artificial intelligence (AI)-based biomaterials and in integrating multi-omics tools to provide patient-tailored therapy. We finally touch on the ongoing limitations and enumerate future directions required to take forward biomaterial-based therapies towards clinical reality.

## 1 Introduction

PH is an idiopathic and potentially life-threatening cardiovascular disease with an elevated pulmonary vascular resistance and pulmonary arterial pressure gradually increasing and eventually causing right heart failure and early death (Galiè et al., 2015; Hassoun, 2021). Despite considerable progress in elucidating the pathophysiological alterations and developing multifaceted therapeutic methods, successful control of the disease is still an imposing clinical challenge and has an estimated 5-year survival rate of only 61.2% (Farber et al., 2015). Present therapies involve vasodilation and blocking pathological vascular growth, but without reversing the causative vascular structure remodeling.

Biomaterials provide an innovative therapeutic concept ideally matched to correct these regenerative deficiencies (Hu et al., 2022; Fakoya et al., 2018). Historically, nanomaterials have been forefront to PH therapeutic endeavors owing to their capacities in controlled release and site-specific delivery of drugs. Biomaterials offer a more integrated approach by mimicking more fully the pulmonary extracellular matrix (ECM), facilitating tissue integration and regenerative signals (Moisa et al., 2024).

The paradigm transition from synthetic nanomaterials to biomaterials is one revolutionary paradigm shift in treatment strategies. Natural polymers, matrixes which are decellularized, and materials from cells contain intrinsic biological signals carrying out tissue repair and regeneration (Khanna et al., 2021; Diksha et al., 2024). Additionally, advances in biomaterial engineering provide the capability to create advanced delivery systems combining biological and synthetic materials to maximize therapeutic effectiveness (Huang and Ding, 2024).

This review critically assesses the current uses and future prospects of biomaterials in therapy against PH in a systematic manner. It first considers the types of biomaterials—such as natural polymers, decellularized matrices, cell-derived materials, and engineered bio-constructs—and how these engage with the pathophysiological processes underlying PH. It further examines their uses in drug delivery, cell therapy, and tissue engineering. The review secondly considers the synergistic union with nanotechnology and shows how this synergy greatly improves therapy efficacy. Thirdly, it considers the important issues surrounding clinical translation, including manufacturing processes and procedures, regulation and requirements, and costs. Those developments offer tremendous promise to researchers and clinicians who are committed to bettering patient outcomes in PH.

## 2 Biomaterials in pulmonary vascular therapy

The use of biomaterials in PH treatment presents a multidimensional solution to vascular repair and regeneration. Four main classes of materials are employed: natural polymers and proteins, decellularized matrix materials, cell-derived products, and engineered bio-constructs. Each class has unique benefits to treating the complicated pathobiology of PH and retains vital characteristics of biocompatibility and physiological significance.

Natural polymers and proteins are the basic building blocks to tissue repair with adjustable mechanical properties and biomimicry capabilities. Decellularized matrices maintain the sophisticated structure and biochemical signals of the *in situ* tissues to stimulate natural regenerative processes. Cell-derived materials tap into the therapeutic value of cellular products without facing the hurdles related to conventional cell therapy. Engineered bio-constructs that occupy the pinnacle of biomaterials engineering incorporate numerous approaches to further tailor therapeutic effectiveness.

**FIGURE 1:**
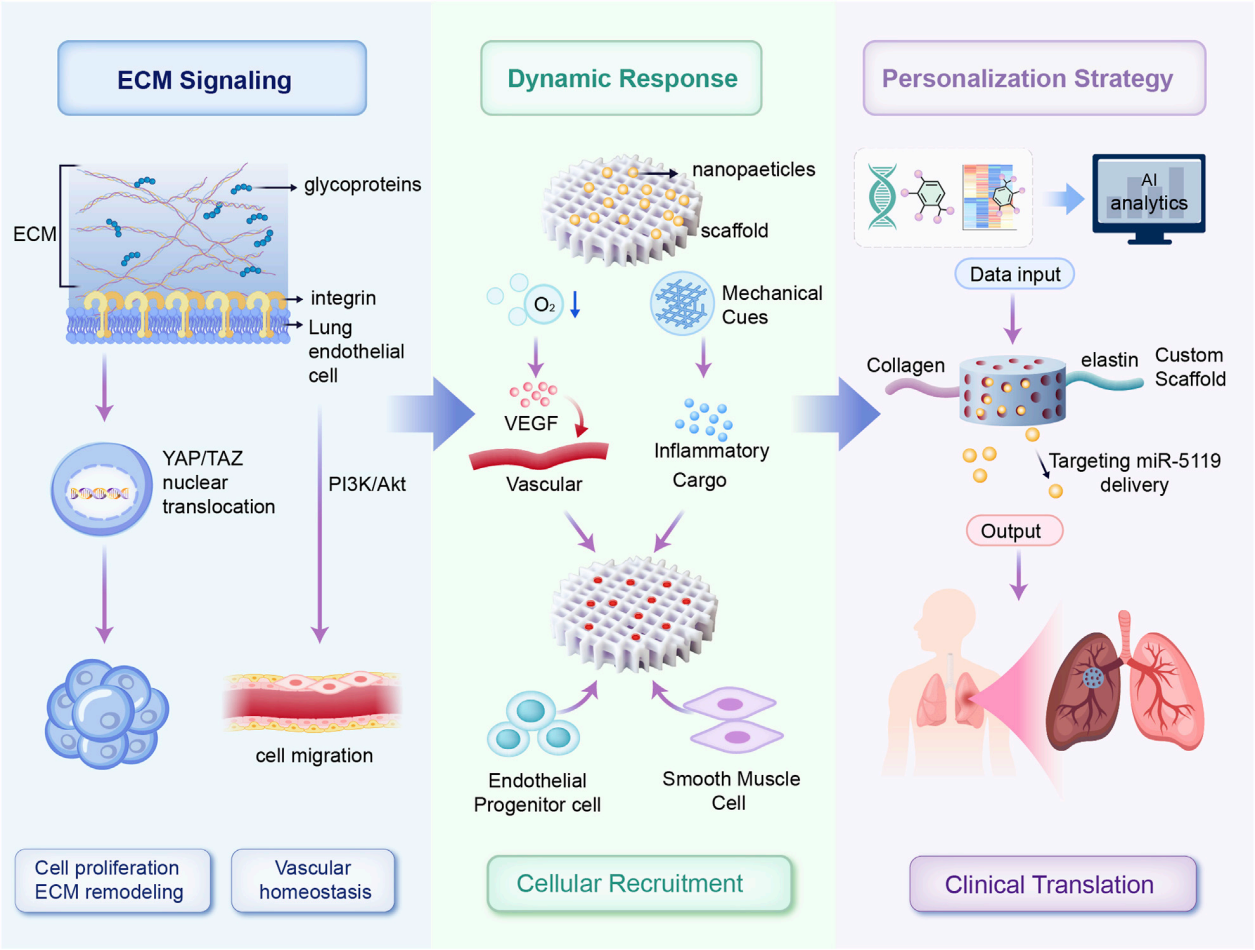
Schematic overview of personalized dynamic biomaterial platforms for vascular regeneration. ECM-derived biochemical cues activate endothelial repair pathways via integrin-mediated YAP/TAZ and PI3K/Akt signaling. Adaptive biomaterial scaffolds respond dynamically to microenvironmental stimuli (hypoxia and mechanical cues), triggering controlled release of therapeutic molecules and recruitment of vascular cells. Multi-omics data integrated through AI-driven analysis enable patient-specific customization of scaffold composition and bioactive payloads, facilitating targeted neovascularization and reduced pathological remodeling in PH.

Biomaterials interact at multiple biological levels, providing structural support, therapeutic delivery, and active modulation of the local cellular and molecular environment essential for vascular repair. Natural polymers, such as collagen and elastin scaffolds, activate integrin-mediated signaling pathways, particularly the PI3K/Akt and ERK1/2 cascades, to promote endothelial cell proliferation, migration, and vascular homeostasis (Lemay et al., 2024). Decellularized matrices preserve key ECM components, such as fibronectin and laminin, which engage β1-integrins to coordinate cytoskeletal remodeling and endothelial stabilization. Cell-derived therapies, particularly exosome-based approaches, modulate hypoxia-inducible factor-1α (HIF-1α) and Runx2 signaling pathways to attenuate pathological vascular remodeling (Zhang et al., 2024). Furthermore, decellularized ECM scaffolds regulate Yes-associated protein/transcriptional co-activator with PDZ-binding motif (YAP/TAZ) signaling by transmitting mechanical cues that control nuclear localization and transcriptional activity, thereby promoting endothelial cell proliferation and reducing vascular remodeling (Jandl et al., 2023; Papavassiliou et al., 2024; Wang et al., 2023). Smart, stimuli-responsive scaffolds promote vascular endothelial growth factor (VEGF)-mediated angiogenesis under hypoxic conditions. Collectively, these mechanisms demonstrate that biomaterials act as bioactive modulators capable of reprogramming pathogenic signaling networks in PH. Building upon these molecular insights, the following sections discuss specific classes of biomaterials and their applications in pulmonary vascular therapy. These mechanisms are schematically illustrated in Figure 1.

### 2.1 Natural polymers and proteins

Natural polymers and proteins, widely used in PH therapy, offer excellent biocompatibility and biomimicry of the extracellular matrix (ECM). Collagen-based materials, abundant in vascular tissues, are particularly versatile and modifiable for tissue engineering applications (Kim et al., 2015). Type I and III collagen, predominant in vascular walls, can be processed into hydrogels and scaffolds that support cell adhesion and migration essential for vascular repair (Lin et al., 2019). Recent studies show that glycosaminoglycan-modified collagen hydrogels enhance vascular tissue repair by regulating protein interactions and promoting endothelial cell growth (Rother et al., 2017). Additionally, collagen-based biomaterials stimulate the proangiogenic functions of CD34^+^ cells through microRNA-21 activation, offering innovative strategies for vascular regeneration (McNeill et al., 2019).

Elastin-derived materials contribute significantly to maintaining vascular compliance and elasticity. Engineered elastin-like polypeptides (ELPs), exhibiting tunable mechanical properties and environmental responsiveness such as temperature-dependent self-assembly (Blit et al., 2012), have demonstrated efficacy in reducing arterial stiffness and supporting pulmonary vascular remodeling (Mahara et al., 2017). Furthermore, ELP-based scaffolds enable minimally invasive delivery and *in situ* formation, providing practical applications in PH therapy (Miao et al., 2005).

Fibrin scaffolds, critical in natural wound healing, also hold unique advantages in pulmonary vascular repair. Derived from patient blood, fibrin scaffolds minimize immunogenicity risks (Anitua et al., 2019). Innovations in fibrin engineering have produced injectable formulations that enable the targeted, sustained delivery of growth factors and genes to affected vessels (Saul et al., 2007). Studies highlight fibrin matrices’ ability to enhance cellular activity, promote tissue regeneration, and improve scaffold mechanical strength and resistance to contraction (Brougham et al., 2015; Thomson, 2013).

### 2.2 Decellularized matrix materials

Decellularized matrix materials provide a biologically relevant scaffold for vascular therapy in PH by preserving the complex biochemical and structural features of native tissues while removing immunogenic cellular components (Ketchedjian et al., 2005). Studies demonstrate that inhalation of solubilized decellularized ECM normalizes alveolar morphology and enhances cellular survival by reducing apoptosis and oxidative damage in preclinical models of acute lung injury (Wu et al., 2017). Moreover, lung-derived decellularized ECM scaffolds support endothelial cell cultures and restore vascular function, offering a robust platform for pulmonary tissue engineering (O'Neill et al., 2013). The combined use of decellularized ECM with therapeutic cells has also demonstrated safety and efficacy in clinical models (Gentile et al., 2020).

Lung-specific ECM materials retain the biochemical and structural characteristics of the pulmonary vascular system while effectively eliminating immunogenic components (Hill et al., 2015). Optimized processing methods ensure the preservation of critical matrix proteins, growth factors, and mechanical properties, while achieving complete decellularization (Kuşoğlu et al., 2023). These materials demonstrate exceptional compatibility with lung endothelial cells, supporting vascular network formation and functionality superior to synthetic alternatives (Özdinç et al., 2023). Decellularized ECM hydrogels maintain cell viability and support tissue regeneration and are an ideal platform for pulmonary tissue engineering and stem cell delivery (Dabaghi et al., 2021).

Moreover, vascular ECM derivatives are responsible for the therapeutic effect by adjusting cellular behavior and tissue permeability. As noted above, these materials modulate important mechanotransduction pathways like YAP/TAZ signaling to enhance vascular remodeling and stabilization of the endothelium (Thenappan et al., 2018; Bertero et al., 2016). Such findings highlight the therapeutic value of ECM-based approaches towards enabling the targeting of anti-PH therapy.

### 2.3 Cell-derived materials

Cellular materials provide an attractive PH therapy approach by tapping into the therapeutic value of cell products with the advantage of avoiding the intricacies related to cell transplantation itself. Biomaterials facilitating the growth and differentiation of induced pluripotent stem cells (iPSCs) are highly promising in PH therapy (Gu et al., 2017). As an instance, iPSC-derived exosomes can prevent pulmonary arterial remodeling by inhibiting the HIF-1α and Runx2 pathways and offer an innovative cellular-therapy-independent approach (Chi et al., 2024). Furthermore, high-density drug screening with iPSC-derived endothelial cells revealed candidates like AG1296, which enhances vascular function and cell survival in PH animal models (Gu et al., 2021). Platforms not only exhibit therapeutic potential but importantly offer insight into the mechanism of PH pathogenesis and individualized intervention opportunities (Campbell et al., 2021).

The stem cell secretome with bioactive molecules such as growth factors, cytokines, and EVs has been found to be an effective therapeutic tool (Bari et al., 2019). The study says that the mesenchymal stem cell-secreted secretome has been shown to decrease pulmonary vascular resistance and enhance right ventricular function in PH models (Muhammad et al., 2020). In particular, MSC-secreted EVs are found to be able to reverse experimental PH by modulating macrophage function and enhancing vascular repair (Klinger et al., 2020). Exosomes derived from MSCs enhance vascular remodeling through VEGF signaling, and this opens up an alternative cell-free therapeutic approach to PH (Sharma et al., 2020). Engineered exosomes provide an attractive tool with which to deliver drugs to specific tissues with high precision in PH. The surface engineering techniques, including conjugation with targeting peptides (e.g., RVG or lung-targeting peptides), dramatically enhance the targeting ability of exosomes to pulmonary vascular endothelial cells (Qiu et al., 2024). Moreover, cargo-loading techniques—including electroporation and exosome–liposome hybridization—enable the efficient delivery of small molecules, nucleic acids, and proteins (Ahmed et al., 2024). Engineered exosomes have demonstrated improved biodistribution, reduced off-target accumulation, and enhanced therapeutic efficacy in preclinical models of pulmonary vascular diseases, underscoring their potential as next-generation personalized delivery vehicles for PH therapy (Huang et al., 2023).

EVs demonstrate exceptional potential in PH treatment due to their ability to target multiple disease pathways simultaneously. Studies show that MSC-derived EVs protect against monocrotaline-induced PH by reducing pulmonary arterial pressure, right ventricular hypertrophy, and vascular remodeling (Blume Corssac et al., 2020). These vesicles, derived from therapeutic cell populations, improve right ventricular function and deliver specific microRNAs that inhibit vascular remodeling and endothelial dysfunction (Wang et al., 2020). Furthermore, the dual role of EVs as both biomarkers and therapeutic agents underscores their transformative potential in PH treatment, bridging diagnostics and therapeutics for more integrated care (Conti et al., 2023).

### 2.4 Engineered bio-constructs

Engineered bio-constructs represent the integration of advanced biomaterials with cutting-edge manufacturing technologies, enabling the creation of complex therapeutic platforms for PH. Tissue-engineered blood vessels (TEBVs) have shown appreciable advancement with the use of variable biomaterials and cell sources. They mimic the mechanical behavior of native vessels and show growth characteristics, vascular integration, and long-term function in pulmonary circulation models. Such developments set the stage for the clinical use of individualized TEBVs (Shinoka et al., 1998). Advances in techniques of decellularization and recellularization expanded the biocompatibility of TEBVs through improved antithrombogenic and antiproliferative characteristics (Quint et al., 2011). Advance in manufacturing techniques has further streamlined the scalability and reproducibility of TEBVs and overcame important hurdles in its clinical translation (Nemeno-Guanzon et al., 2012).

Bioprinted vascular structures provide an innovative means to produce patient-specific therapeutic solutions. State-of-the-art bioprinting technologies provide control and customization options regarding material composition and architecting to construct complex vascular networks. The structures are usually equipped with smart release systems that react to biological signals effectively inducing angiogenesis and enhancing tissue integration (Cui et al., 2016). High-resolution printing makes it possible to custom-tailor geometric parameters with a focus on maintaining long-term cell viability and enhancing clinical feasibility (Foresti et al., 2020). In addition to this, scaffold-free bioprinting methods have been used to build small-diameter and highly branched vascular networks with renewed prospects in tissue engineering (Norotte et al., 2009).

Smart bio-scaffolds are designed with adaptive components to assist with vascular regeneration within the developing pulmonary environment (Thottappillil and Nair, 2015). Chemotactic smart scaffolds with VEGF promote the formation of capillaries and recruit stem cells to support individualized vascular repair (Hokugo et al., 2015). New bio-manufacturing methods have led to the creation of scaffolds that can replicate the dynamic aspects of native ECM. Such systems adjust their properties based on local tissue needs and maximize function and therapeutic efficacy (Ahadian and Khademhosseini, 2018). Functionalization strategies on scaffolds, such as the conjugation of cell adhesion peptides (i.e., RGD motifs), enhance cell attachment and vascularization of endothelial cells (Liguori et al., 2024). Growth factor incorporation into biomaterial matrices, like VEGF and basic fibroblast growth factor (bFGF), enhances vascularization and tissue regeneration. Moreover, surface bioactive coatings derived from ECM proteins, such as fibronectin and laminin, facilitate cellular integration and functional recovery (Liu et al., 2024). Advanced functionalization approaches—including smart polymers and nanostructured coatings—enable the fabrication of adaptive scaffolds tailored to the evolving pulmonary microenvironment.

### 2.5 Comparison to approved nanomedicines

Although approved nanomedicine-based therapies for PH—such as prostacyclin-loaded liposomes and nanoparticle-based vasodilator systems—focus on improving bioavailability and targeting, biomaterials offer distinct regenerative advantages. Bio-derived platforms demonstrate superior biocompatibility, reduced immunogenicity, and active participation in tissue regeneration. Unlike conventional nanomedicines, which often function as passive carriers, biomaterials mimic the ECM microenvironment to promote endothelial repair and vascular remodeling (Abdelhamid et al., 2024; Parida and Raheem, 2025). Furthermore, their biodegradability and tunable mechanical properties enhance therapeutic integration and durability, offering a regeneration-oriented and translationally relevant strategy for future PH management (Krishnan et al., 2024).

### 2.6 Comparative efficacy and limitations of biomaterials versus current pharmacological therapies in PH

Current pharmacological treatments for PH—including prostacyclin analogs, endothelin receptor antagonists, and phosphodiesterase type 5 (PDE5) inhibitors—aim to promote vasodilation, inhibit smooth muscle proliferation, and improve hemodynamics (Rasheed et al., 2024). Although these therapies significantly alleviate symptoms and improve short-term survival, they do not address the underlying structural vascular remodeling that drives disease progression (Fujiwara et al., 2025). Moreover, long-term pharmacotherapy often causes systemic side effects, offers limited reversal of vascular lesions, and requires continuous administration (Tang et al., 2024).

**FIGURE 2:**
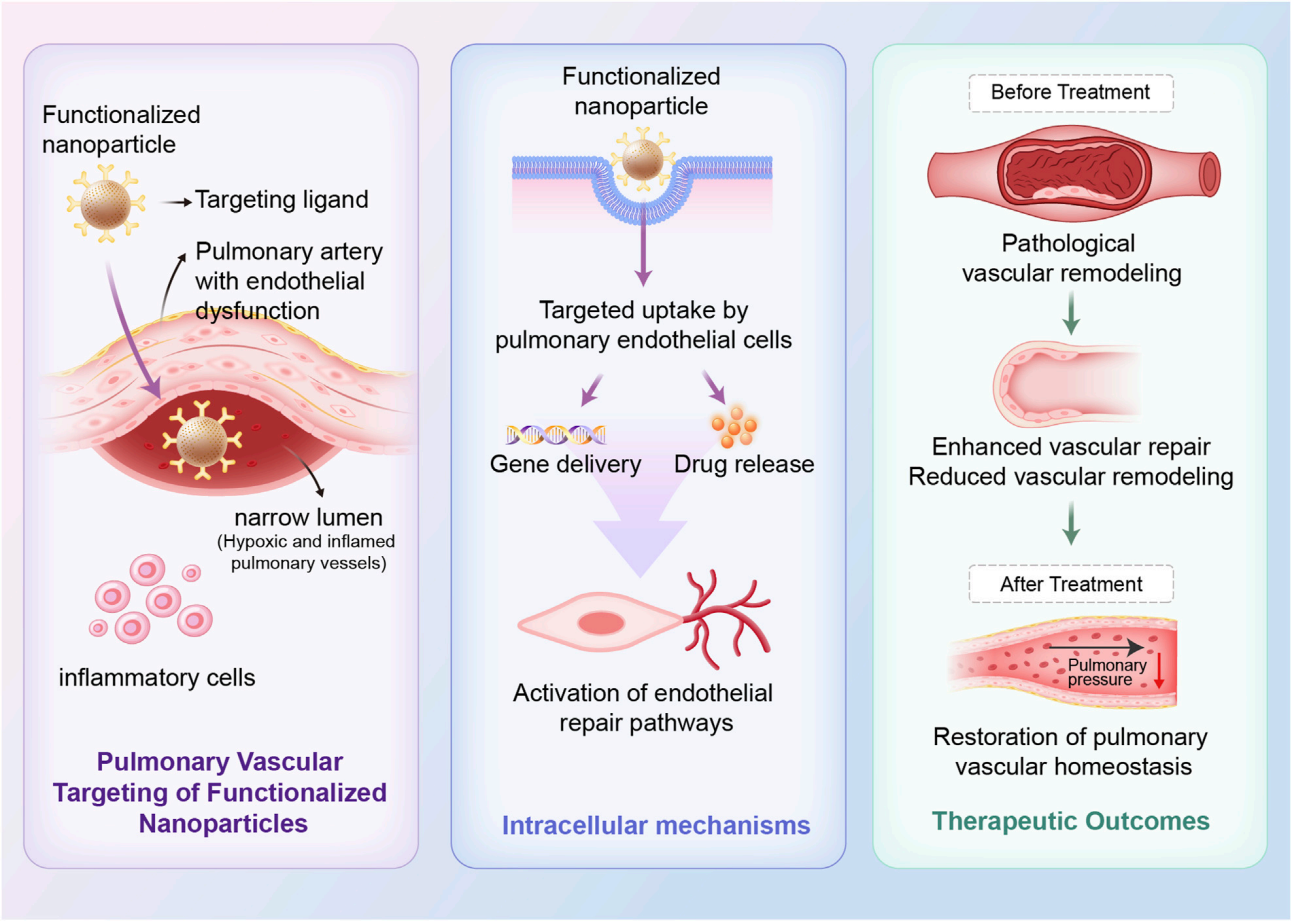
Therapeutic mechanisms of bio-nano interfaces for restoring pulmonary vascular homeostasis in PH. Targeted nanoparticles, conjugated with endothelial-specific ligands, preferentially accumulate in pulmonary arteries with endothelial dysfunction. Following targeted uptake by pulmonary endothelial cells, nanoparticles enable intracellular release of therapeutic agents, including nucleic acids and anti-inflammatory agents. This process supports vascular repair and reduces vascular remodeling, ultimately restoring pulmonary vascular homeostasis.

In contrast, biomaterial-based therapies directly modulate the pulmonary microenvironment, promote endothelial repair, and deliver regenerative or immunomodulatory signals. Biomaterials such as extracellular matrix scaffolds, engineered nanoparticles, and exosome-based carriers can guide cellular behavior and facilitate tissue repair to promote vascular healing (Capella-Monsonís et al., 2024). The combination of engineered nanomaterials with EVs enables localized, sustained therapeutic delivery while minimizing systemic toxicity (Jiang et al., 2025). Furthermore, the ability to customize biomaterials according to patient-specific pathological features offers a significant advantage for advancing precision medicine in PH. The therapeutic effects and vascular remodeling processes mediated by these biomaterials are depicted in Figure 2.

Nevertheless, biomaterial-based therapies face distinct challenges, including the complexity of large-scale manufacturing, potential immunogenicity, challenges in achieving sustained pulmonary targeting, and stringent regulatory hurdles. Therefore, while biomaterials represent a highly promising Frontier, their integration with existing therapies or use as adjuncts may provide synergistic benefits rather than serving as direct replacements (Kashizaki et al., 2024).

### 2.7 Personalized therapeutic strategies

Biomaterial-based personalized therapies represent a significant advancement in the management of PH. Unlike traditional “one-size-fits-all” designs, patient-specific biomaterial development integrates patient-specific genetic, transcriptomic, epigenetic, and immunological data.

This personalized approach enables precise tailoring of biomaterial properties, including composition, surface functionalization, mechanical stiffness, and bioactive cargo, to match each patient’s pathological phenotype and vascular remodeling status. For example, scaffold degradation rates and mechanical properties can be adjusted to accommodate patient-specific pulmonary hemodynamics (Chen H. et al., 2024), while bioengineered EVs can be customized to enhance selective delivery and improve vascular remodeling, with cargos such as miR-5119 targeting patient-specific pathological pathways (Zhu et al., 2024).

Moreover, omics-guided design, leveraging genomics, proteomics, metabolomics, and transcriptomics, allows for systematic customization of scaffold characteristics and therapeutic payloads in PH treatment. Advances in single-cell omics technologies have enabled unprecedented dissection of the cellular ecosystem in PH, revealing novel engineering targets for biomaterial-based interventions tailored to specific patient populations (Rafikov et al., 2025). AI-driven predictive models optimize this process by integrating omics data, modeling complex interactions, and predicting ideal biomaterial designs (Nam et al., 2024).

Collectively, these strategies signify a transformative shift toward biomaterials that not only provide structural support but also dynamically adapt to each patient’s evolving pathological landscape, thereby advancing the paradigm of precision medicine in PH therapy. Emerging technologies, including AI-driven optimization and multi-omics integration, are expected to further revolutionize biomaterial personalization (discussed further in Section 5).

Table 1 was newly created to systematically summarize all discussed biomaterials, including material type, released molecules, applications, results obtained, and references.

**TABLE 1:** Summary of biomaterials for pulmonary hypertension therapy.

Material type	Molecules released	Applications	Results obtained	References
Collagen-based hydrogels	microRNA-21 stimulation	Vascular regeneration, tissue repair	Promotes endothelial cell proliferation, improves repair	Kim et al. (2015), Lin et al. (2019), Rother et al. (2017), McNeill et al. (2019)
Elastin-like polypeptides (ELPs)	None (structural)	Restore vascular elasticity	Reduces stiffness, supports remodeling	Blit et al. (2012), Mahara et al. (2017), Miao et al. (2005)
Fibrin scaffolds	VEGF, growth factors	Localized drug delivery, vascular repair	Enhances cell survival and tissue integration	Anitua et al. (2019), Saul et al. (2007), Brougham et al. (2015), Thomson (2013)
Decellularized lung ECM	Native ECM proteins	Lung tissue engineering, vascular regeneration	Normalizes alveolar morphology, improves endothelial function	Hill et al. (2015), Kuşoğlu et al. (2023), Özdinç et al. (2023), Dabaghi et al. (2021)
Decellularized vascular ECM	Retention of YAP/TAZ pathway regulators	Remodeling control, endothelial stabilization	Activates YAP/TAZ signaling, reduces dysfunction	Thenappan et al. (2018), Bertero et al. (2016)
iPSC-derived exosomes	microRNAs (e.g., anti-HIF-1α, anti-Runx2)	Non-cell therapy, vascular repair	Suppresses vascular remodeling	Chi et al. (2024), Gu et al. (2021), Campbell et al. (2021)
MSC-derived EVs	VEGF, cytokines	Vascular regeneration, immune modulation	Reduces RV hypertrophy, modulates macrophages	Klinger et al. (2020), Sharma et al. (2020)
Engineered exosomes	Nucleic acids, proteins	Precision drug delivery	Enhances biodistribution and targeting	Qiu et al. (2024), Ahmed et al. (2024), Huang et al. (2023)
Tissue-engineered blood vessels (TEBVs)	None (structural)	Vascular reconstruction	Emulates vessel properties, supports patency	Shinoka et al. (1998), Quint et al. (2011), Nemeno-Guanzon et al. (2012)
Bioprinted vascular scaffolds	VEGF, angiogenic factors	Customized vascular therapies	Enhances angiogenesis and integration	Cui et al. (2016), Foresti et al. (2020), Norotte et al. (2009)
Smart scaffolds	VEGF, bioactive agents	Targeted regenerative scaffolds	Adapts scaffold properties to microenvironment	Thottappillil and Nair (2015), Hokugo et al. (2015), Ahadian and Khademhosseini (2018), Liguori et al. (2024), Liu et al. (2024)
Magnetic nanoparticle-embedded ECM	None (magnetically targeted delivery)	Magnetically targeted vascular therapy	Magnetic-guided vascular targeting	Thottappillil and Nair (2015), Hokugo et al. (2015), Ahadian and Khademhosseini (2018), Liguori et al. (2024), Liu et al. (2024)
Mesoporous silica nanoparticles	Small-molecule drugs	Controlled drug release	Stimuli-triggered drug release	Thottappillil and Nair (2015), Hokugo et al. (2015), Ahadian and Khademhosseini (2018), Liguori et al. (2024), Liu et al. (2024)
PEGylated nanoparticle composites	Gene payloads (e.g., plasmids)	Gene therapy delivery	Efficient pulmonary targeting, sustained gene expression	Thottappillil and Nair (2015), Hokugo et al. (2015), Ahadian and Khademhosseini (2018), Liguori et al. (2024), Liu et al. (2024)
Stimuli-responsive carriers	Hypoxia-/pH-sensitive drugs	Dynamic drug delivery	Responsive drug delivery in hypoxic environment	Thottappillil and Nair (2015), Hokugo et al. (2015), Ahadian and Khademhosseini (2018), Liguori et al. (2024), Liu et al. (2024)
Inhalable biomaterial formulations	PDE5 inhibitors, prostacyclin analogs	Localized pulmonary drug delivery	Improves lung deposition, reduces systemic exposure	Thottappillil and Nair (2015), Hokugo et al. (2015), Ahadian and Khademhosseini (2018), Liguori et al. (2024), Liu et al. (2024)
Spray-drying techniques	Anti-inflammatory agents, ROCK inhibitors	Targeted inhalation therapy	Optimized aerosolization and targeting	Thottappillil and Nair (2015), Hokugo et al. (2015), Ahadian and Khademhosseini (2018), Liguori et al. (2024), Liu et al. (2024)

Table 2 delineates the key molecular pathways modulated by these biomaterials, along with their associated biological effects and supporting references in the context of PH therapy.

**TABLE 2:** Key molecular pathways associated with biomaterial-based therapies in pulmonary hypertension.

Material type	Pathways modulated	Biological effects	References
Natural polymers	PI3K/Akt signaling, ERK1/2 signaling, β1-integrin-mediated signaling, microRNA-21 activation	Promotes endothelial cell proliferation, migration, and vascular homeostasis; Stimulates endothelial activation; Facilitates cytoskeletal remodeling and endothelial stabilization; Enhances proangiogenic function of CD34^+^ cells	Lemay et al. (2024), McNeill et al. (2019)
Decellularized ECM	YAP/TAZ signaling, β1-integrin-mediated signaling	Promotes vascular smooth muscle cell remodeling and endothelial stabilization; Coordinates cytoskeletal remodeling and vascular repair	Jandl et al. (2023), Papavassiliou et al. (2024), Wang et al. (2023), Thenappan et al. (2018), Bertero et al. (2016)
MSC-derived exosomes	HIF-1α signaling, Runx2 signaling, VEGF signaling	Attenuates pathological vascular remodeling; Suppresses vascular smooth muscle cell proliferation; Enhances vascular remodeling and angiogenesis	(Zhang et al., 2024; Sharma et al., 2020)

## 3 Integration of biomaterials with therapeutic strategies

Integrating biomaterials with existing therapeutic modalities has significantly amplified treatment efficacy in PH. This synergy is particularly evident in three interconnected strategies: drug delivery applications, cell therapy enhancement, and gene therapy. Biomaterials not only serve as physiologically relevant delivery platforms but also actively improve treatment outcomes through biocompatibility, tissue-specific interactions, and sustained therapeutic effects (Wang et al., 2021).

Unlike passive carriers, biomaterials actively participate in therapeutic processes. For instance, endothelial progenitor cell-based therapies, coupled with biomaterials for targeted gene delivery, have shown potential to restore pulmonary vascular health, opening novel avenues for combating PH (Gaine and Gomberg-Maitland, 2007). In gene therapy, biomaterials safeguard genetic material during delivery, ensuring prolonged expression while maintaining cell accessibility. All these integrative methods are evidence of advances in the materials sciences and increased knowledge about the pathophysiology of PH and are leading towards more individualized and holistic treatment approaches.

Additionally, biomaterials support the creation of adaptive therapeutic systems that can adjust to the dynamic state of pulmonary vascular diseases. Stimuli-responsive biomaterials are able to alter physical and chemical properties when environmental conditions change, ensuring optimal drug release and structure adaptability to enhance clinical outcomes (Tapeinos et al., 2022). Combining biocompatible natural resources with state-of-the-art delivery technologies in this approach leads to efficient therapeutic platforms that are able to target delivery and sustain cellular function and tissue repair (Facklam et al., 2020). The integration with high-precision manufacturing techniques enhances scalability, uniformity, and therapeutic effectiveness.

### 3.1 Drug delivery applications

The pairing with drug delivery systems is an important advancement in PH treatment that increases bioavailability, accuracy, and long-term delivery and is physiologically compatible. Biomaterials provide one attractive solution to maximize existing drug treatments’ delivery and therapeutic effectiveness in PH, including prostacyclin analogs and PDE5 inhibitors. Entrapping these drugs in biomaterial-modified hydrogels, microspheres, or nanoparticles allows for steady-state release with local efficacy and therefore lowers the risk of adverse effects from systemic exposure (Nakamura et al., 2019). As an illustration, prostacyclin analogs entrapped in fibrin hydrogels provide prolonged pulmonary vasodilation and more efficient vascular repair in preclinical models (Akagi et al., 2016). Likewise, controlled release from disease-localizing biodegradable polymer carriers like solid lipid nanoparticles will deliver PDE inhibitors and reduce dosing intervals (Makled et al., 2017). Therefore, drug delivery platforms aided by biomaterials are an uplifting complementary advancement to existing pharmacologic therapy in PH.

Biomaterial-based controlled release systems have shown better drug delivery characteristics. Natural polymer carriers like albumin and hyaluronic acid support drug release over an extended period, thus maximizing bioavailability and minimizing dosing intervals (Fenton et al., 2018). Injectable collagen microspheres, for instance, sustain drug concentrations within regions of interest to effectively modulate the multifaceted PH pathology (Nagai et al., 2010).

Protein-drug conjugates offer yet another novel drug delivery mechanism. They make use of natural proteins to serve as carriers during site-specific delivery and stabilize drugs. Examples include protein-drug conjugates with carboxybetaine polymers with zwitterionic characteristics in improving pulmonary bioavailability to provide novel treatment options for PH (Tsao et al., 2020).

### 3.2 Cell therapy enhancement

Biomaterials have transformed cell therapy in PH by developing supportive environments to promote cell survival, function, and integration with host tissue.

Cellular retention and viability are greatly enhanced by natural polymer-based delivery matrices through mechanical stabilization and biomimic patterns. RGD-modified hyaluronic acid hydrogels, for instance, promote the survival and migration and organization into vascular networks of endothelial cells and show candidacy in vascular repair (Bidarra et al., 2011). Cell viability and interaction with the ECM are similarly enhanced by chitosan-fibrin microspheres and provide an ideal minimally invasive route for cell delivery (Chen et al., 2011).

Survival-enhancing materials include bioactive signals to provide support to therapeutic cells’ functionality. Matrices with added growth factors like EGF and VEGF stimulate vascular regeneration by enhancing survival and vascularization in endothelial and smooth muscle cells (Lequoy et al., 2016). Such systems include nanotechnology like porous scaffolds to release growth factors under controlled conditions to functionally emulate the ECM and form protective microenvironments (Wang et al., 2017). New small-molecule adhesives and ECM coatings enhance cellular adhesion and regeneration and are economical means of improving therapy (Mitchell and Lo, 2023).

### 3.3 Gene therapy platforms

The integration of biomaterials with gene therapy has opened up new avenues in treating PH on the level of molecules and confronting conventional gene-delivery limitations.

Biomaterial-based carriers provide safer and more stable options compared to viral vectors with equally efficient gene transfer capabilities. For example, the “Sleeping Beauty” transposon system has proved to reduce pulmonary arterial pressure and vascular remodeling through non-viral gene delivery (Liu et al., 2006). Such systems shield nucleic acids against degradation by nucleases, are more stable, and exhibit controlled release capabilities by providing alternative passages to drugs and biological molecules by evading nuclease degradation and poor permeability through cell membranes (Zhuang and Cui, 2021). In comparison with traditional gene transfer mediated by viral vectors, biomaterial-assisted platforms provide important advantages with respect to safety, including negligible risk of insertional mutagenesis, lower immunogenicity, and increased scalability in production (Yu et al., 2023). Although viral vectors promote greater transfection efficacy, they are linked to issues with long-term safety and regulatory considerations (Huang et al., 2024). In contrast to these, although through current developments biomaterial-based carriers are realizing only modest transfection rates, these are being quickly improved through embedding targeting ligands and efficient nucleic acid condensation methods. Such advancements place biomaterial-assisted systems in an emerging and more secure role to perform gene therapy in PH (Wang H. et al., 2022).

Hybrid biomaterial platforms have been created to provide long-term gene expression in target tissues as well. Recent developments comprise systems that involve conjugation of natural polymers to DNA-binding domains to promote localized delivery and long-term therapeutic action (Yu et al., 2023).

Additionally, combination therapies with genes and drugs and biomaterials have been found to produce synergistic advantages. Angiogenic genes and anti-inflammatory drugs in matrices are more efficient in repairing pulmonary vasculature in preclinical experiments compared to single-modality treatments (Reynolds, 2011).

The successful convergence with conventional therapeutic approaches highlights the revolutionary nature of PH treatment with biomaterials. They offer solutions to several aspects of the disease without compromising on biocompatibility and targeting, creating the foundation for more individualized, efficient, and responsive therapeutic platforms.

## 4 Use of bio-nano interfaces in PH treatment

The intersection of nanotechnology and biomaterials is itself a paradigm revolution that enables the creation of very high-precision, biocompatible, and multifunctional therapeutic platforms. By integrating the high-precision and advanced functionality of nanotechnology with the bioactivity and biocompatibility of biomaterials, bio-nano interfaces have become very powerful therapeutic tools (Segura-Ibarra et al., 2018). Two major strategies are used to realize bio-nano interfaces: the use of biomaterial-nanoparticle composites and hybrid delivery systems that surpass conventional limitations of standalone nanomaterials or biological counterparts.

### 4.1 Biomaterial-nanoparticle composites

The union of biomaterials and nanoparticles is revolutionary in PH therapy by harnessing the stability offered by biomaterials and the targeting ability of nanoscale structures to maximize therapeutic efficacy. The integration fills some major gaps like short half-life drugs and poor targeting specificity with more efficient disease intervention (Mosgoeller et al., 2012). New generations of biomaterial-nanoparticle composites involve the introduction of functional nanoparticles with the beneficial aspects of biological matrices (Freeman, 2021). Specifically, the PEG-modified composite nanoparticles were found to decrease toxicity and enhance the efficacy of pulmonary-targeted gene delivery with remarkable accumulation in PH models (Kolte et al., 2017). Additionally, surface engineering like hydrophobicity modulation improves pulmonary biocompatibility to provide improved and more secure therapeutic delivery (Jones et al., 2014).

Besides mechanical reinforcement, these composites possess dynamic therapeutic function. Decellularized matrices with incorporated magnetic nanoparticles provide target vascular delivery under external magnetic fields without sacrificing the regenerative function of natural ECM (Gil and Mano, 2014). Likewise, mesoporous silica nanoparticles bypass anatomical and physiological barriers to pulmonary drug delivery by allowing controlled, stimulus-sensitive release, effectively mitigating solubility issues (Garcia-Fernandez et al., 2021). Chemistry on the surface of biomaterial–particle composites is critical to controlling cellular interaction, uptake, and overall therapeutic response. Principal surface characteristics—surface charge, hydrophobicity, ligand density, and nanoscale topography—control protein adsorption, intracellular traffic, and immunity recognition. As an illustration, surface-modified nanoparticles with neutral or mildly negative surface charge and with added polyethylene glycol (PEG) show enhanced pulmonary circulation times and decreased macrophage-mediated clearance and thus superior pulmonary gene and drug delivery (Tehrani et al., 2022). Further, ligand functionalization to pulmonary endothelial markers under conditions of hypoxia allows site-specific tissue accumulation and drug retention (Shah et al., 2012). Therefore, careful surface engineering is necessary to optimize the biological function of the bio–nano interface in therapy against PH.

### 4.2 Hybrid delivery systems

Hybrid bio-nano delivery systems have significantly improved the precision and efficacy of PH treatment by combining nanoscale delivery tools with bio-recognition elements. These systems leverage stimuli-responsive designs to achieve dynamic targeting of diseased tissues, addressing the complex pathophysiology of PH. For instance, nanocarrier systems engineered to anchor onto E-selectin expressed by pulmonary endothelial cells under hypoxic conditions have demonstrated precise drug delivery to hypoxic pulmonary arterial cells, enhancing therapeutic retention and mitigating vascular remodeling. Innovations in microfluidics further advance the fabrication of these hybrid systems, enabling precise control of nanoparticle size, shape, and surface modifications (Chen et al., 2022). Microfluidics also facilitates the scalable production of lipid nanoparticles and extracellular vesicle mimetics, offering new possibilities for targeted drug delivery (Piunti and Cimetta, 2023).

The development of multifunctional bio-nano platforms has expanded the scope of PH treatment. For example, microRNA-based nanotherapeutics not only enhance stability and localization but also integrate diagnostic and therapeutic functions, driving treatment strategies toward precision medicine (Carregal-Romero et al., 2020). Additionally, responsive nanocarriers and biomimetic structures, such as gene delivery systems targeting vascular endothelium, demonstrate remarkable potential in achieving real-time adaptation to disease progression, addressing the complex challenges of PH (Yuan et al., 2023).

The integration of biomaterials and nanotechnology represents a fundamental shift in PH treatment paradigms, enabling the development of personalized, responsive, and highly effective therapeutic platforms. As our understanding of bio-nano interactions deepens and advanced manufacturing technologies evolve, bio-nano interfaces are expected to play an increasingly critical role in addressing the challenges of pulmonary vascular diseases. Continued innovation in this field holds the promise of more precise, adaptive, and multifunctional therapeutic systems capable of improving patient outcomes.

### 4.3 Stimuli-responsive biomaterials for PH therapy

Stimuli-responsive biomaterials offer an innovative strategy for addressing the dynamic microenvironment of PH. Hypoxia-responsive carriers are engineered to release therapeutic agents under low oxygen tension, enabling selective targeting of diseased pulmonary arteries while minimizing systemic exposure. Similarly, pH-sensitive nanoparticles are designed to discharge their payloads within the acidic, inflammatory microenvironment characteristic of advanced PH, thereby enhancing delivery specificity (Gaddimath et al., 2024; Gao et al., 2025). Additionally, redox-responsive materials that exploit the oxidative stress present in PH vasculature provide an added layer of control for site-specific drug release (Parvin et al., 2025). The integration of such stimuli-responsive platforms enables real-time adaptation to disease progression, improves therapeutic efficacy, and reduces off-target effects, making them particularly promising for personalized PH treatment strategies (Chen X. et al., 2024).

### 4.4 Potential risks of bio-nano interfaces and mitigation strategies

Despite the important therapeutic promise of the bio–nano interface, specific risks of its clinical application in PH must be carefully mitigated. One of the main concerns is the accumulation of nanoparticles in non-target organs such as the liver, spleen, and kidneys, potentially causing long-term toxicity and organ damage. Moreover, bio-nanomaterials may trigger adverse immune responses, including complement activation, macrophage uptake, and inflammatory reactions, which could worsen vascular damage and promote pathological vascular remodeling (Li et al., 2024; DeVaughn et al., 2024).

Several approaches have been developed to address these risks. Surface modification of nanoparticles with biocompatible polymers, like PEGylation, can extend circulation time, reduce recognition by the mononuclear phagocyte system, and lower immunogenicity (Elsayed, 2024). The use of biodegradable components, such as poly (lactic-co-glycolic acid) (PLGA) and lipid-based carriers, further allows for controlled degradation and clearance, helping to reduce the risk of long-term accumulation (Castro et al., 2025). Targeting ligands, such as antibodies or peptides specific to pulmonary endothelial cells, can be conjugated to the nanoparticle surfaces to improve targeting accuracy and minimize systemic exposure (Liu et al., 2025). Additionally, combining stealth properties with alternative coatings, like poly (N-vinylpyrrolidone) derivatives, offers innovative methods to modulate immune responses and enhance nanoparticle biocompatibility (Toussaint et al., 2024). Addressing these challenges is crucial for ensuring the safety, efficacy, and feasibility of bio-nano therapies in PH.

Targeted nanoparticles, conjugated with endothelial-specific ligands, preferentially accumulate in pulmonary arteries with endothelial dysfunction. Following targeted uptake by pulmonary endothelial cells, nanoparticles enable intracellular release of therapeutic agents, including nucleic acids and anti-inflammatory agents. This process supports vascular repair and reduces vascular remodeling, ultimately restoring pulmonary vascular homeostasis.

## 5 Emerging directions: AI-enhanced biomaterial platforms and multi-omics personalized therapy for PH

PH is a complex, multifactorial disease characterized by dynamic vascular remodeling, inflammation, and right ventricular dysfunction. Although traditional biomaterial-based approaches have demonstrated promise, they face challenges in achieving truly precise, patient-specific therapeutic outcomes. Recent advances combining AI-driven analytics and multi-omics profiling offer transformative pathways for innovating biomaterial design tailored to patient-specific pulmonary microenvironments (Han and Wu, 2024). These platforms generate predictive models informed by comprehensive omics data, enabling the dynamic tailoring of biomaterial properties (Mozafari, 2025). This integration fosters the creation of precision-engineered scaffolds, advancing the paradigm of personalized PH therapy (Tambi et al., 2024).

### 5.1 AI-driven biomaterial design

AI and machine learning (ML) algorithms are revolutionizing the discovery and optimization of biomaterials. In PH therapy, AI enables rapid screening and prediction of material properties—such as biocompatibility, degradation rates, mechanical strength, and drug release profiles—by analyzing large experimental datasets. Advanced AI models, including generative adversarial networks (GANs) and reinforcement learning, are being used to design polymeric scaffolds, nanoparticles, and hydrogels with enhanced therapeutic functions. For example, AI-assisted optimization can adjust nanoparticle surface chemistry to maximize endothelial cell uptake and minimize immune clearance. Additionally, deep learning models can predict material–cell interactions and simulate therapeutic efficacy based on historical experimental data, which speeds up the development of personalized regenerative therapies for PH (Shiri et al., 2025).

### 5.2 Multi-omics integration for personalized biomaterial therapy

Integrating multi-omics approaches—such as genomics, transcriptomics, proteomics, and metabolomics—offers a holistic view of the molecular mechanisms driving PH progression. These datasets inform the customization of biomaterial-based therapies according to individual patient disease phenotypes and molecular profiles. For instance, single-cell RNA sequencing (scRNA-seq) can guide the strategic incorporation of growth factors, cytokines, or nucleic acids into biomaterial platforms. Proteomic profiling of EVs identifies disease-specific biomarkers, informing the rational design of exosome-inspired delivery systems. Moreover, metabolomic insights enable the engineering of stimuli-responsive biomaterials that release therapeutics in response to patient-specific metabolic alterations such as hypoxia-induced acidosis (Xin et al., 2024; Abdelaziz et al., 2024). Future research should prioritize building comprehensive biomaterial design frameworks that incorporate real-time omics profiling to adapt therapies dynamically throughout the disease course.

## 6 Clinical translation and future perspectives

Many biomaterial-based strategies have demonstrated promising therapeutic efficacy in preclinical models of PH, including modulation of vascular remodeling, promotion of endothelial repair, and targeted drug delivery.

However, despite these advances, comprehensive reviews confirm that no biomaterial-based therapy has yet entered clinical evaluation specifically for PH. Current clinical investigations primarily focus on small-molecule therapies, monoclonal antibodies, or cell-based interventions, rather than scaffold- or matrix-based approaches (Pradhan et al., 2024). This gap underscores that the clinical translation of biomaterials in PH remains at an early stage. To advance biomaterial-based therapies into human studies, several key challenges must be addressed, including the development of scalable and reproducible manufacturing processes, assurance of biocompatibility and batch-to-batch consistency, and successful navigation of complex regulatory pathways (Gorantla et al., 2024). The transition of biomaterials from laboratory discoveries to clinical applications in the treatment of PH presents numerous challenges. Chief among these are achieving scalable production while preserving bioactivity and ensuring compliance with clinical and regulatory standards. For example, additive manufacturing techniques offer significant potential for on-demand customization of biomaterials but face complexities in achieving large-scale production (Bose et al., 2018). Integrating precision biomaterials with biofabrication technologies allows for the simulation of dynamic tissue changes associated with PH and other pulmonary diseases. By aligning manufacturing approaches with disease-specific needs, these strategies address scalability and regulatory barriers while fostering the development of personalized therapies (Bailey et al., 2019).

The potential immunogenicity and long-term *in vivo* stability of biomaterials remain critical considerations for their clinical translation. Strategies to mitigate immune responses include the use of autologous cell-derived materials, surface modification with zwitterionic polymers, and dynamic covalent crosslinking techniques that preserve scaffold structure while minimizing inflammatory reactions (Wang et al., 2024; Pan et al., 2024). In addition, biomaterials engineered with tunable degradation kinetics retain their mechanical strength over extended periods, meeting the demands of pulmonary tissue remodeling (Wang S. et al., 2022). Emerging technologies like dynamic covalent chemistry and biomimetic cross-linkers provide further means of enhancing scaffold longevity and biocompatibility, improving therapeutic outcomes.

The regulatory environment for PH therapy with biomaterials accommodates the special complexities of these new platforms. Clinical applications require overcoming both manufacturing and regulatory hurdles, such as validating the safety and efficacy of multi-component biological products. Risk governance for nano-biomaterials stresses systematic evaluation of patient and environmental risks, ensuring benefits throughout the product life cycle (Giubilato et al., 2020). Moreover, new inhalation therapies emphasize the need for sophisticated drug delivery systems meeting rigorous regulatory standards for reproducibility and clinical compliance (Acosta et al., 2021).

Inhalable biomaterial-formulated therapies are a promising strategy for localized PH treatment, delivering therapeutics directly to the lungs with minimal systemic side effects. Advances in nanoparticle aerosols, hydrogel microdroplets, and liposomal dry powder inhalers have demonstrated both feasibility and clinical potential (Ruiz et al., 2024). Biomaterials like chitosan, hyaluronic acid, and PLGA have been developed into inhalable carriers with enhanced deposition efficiency, stability during aerosolization, and excellent biocompatibility (Albetawi, 2023). These technologies not only improve patient compliance through noninvasive administration but also optimize drug concentrations at the target site, offering the potential to transform PH management strategies.

The clinical implementation of biomaterial-based therapies provides valuable insights while highlighting critical areas for further improvement. Early clinical trials demonstrate that careful patient selection and personalized treatment plans are essential for optimizing therapeutic outcomes (Galiè et al., 2004). Tissue-informed biomaterials and cell-regenerative therapies have shown considerable promise in improving outcomes for PH patients. However, these approaches necessitate specialized training for healthcare providers in handling and managing biomaterials, creating a need for standardized protocols and procedural guidelines to support widespread adoption (Campbell et al., 2021; Foster et al., 2014).

Economic considerations also pose challenges for biomaterial-based therapies, but their potential for long-term value is evident. Although production costs for these advanced therapies are higher compared to traditional medications, their superior therapeutic efficacy may offset initial expenses (Pashuck and Stevens, 2012). For instance, microRNA-based nanotherapeutics, while costly, have demonstrated remarkable precision and efficiency, leading to growing acceptance within healthcare systems (Carregal-Romero et al., 2020). Advances in production methods and the integration of personalized medical strategies are gradually overcoming economic and implementation barriers, paving the way for broader adoption of these platforms in PH treatment.

From a health economics perspective, although biomaterial-based interventions initially entail higher production costs compared to conventional small-molecule therapies, they may offer superior long-term cost-effectiveness. By slowing disease progression, reducing hospitalization rates, and minimizing treatment-related complications, regenerative biomaterial therapies have the potential to substantially lower overall healthcare expenditures (Han et al., 2022). Advances in scalable biomanufacturing technologies and modular design strategies, such as spray-drying techniques, are expected to further reduce production costs and enhance clinical accessibility (Muralidharan et al., 2021). Moreover, localized delivery systems and inhalable biomaterial formulations (e.g., dry powder inhalers) provide cost-effective solutions for resource-limited settings, improving patient adherence and expanding access to PH (PH care on a global scale (Cao et al., 2024).

Looking to the future, biomaterials are expected to play an increasingly vital role in PH therapy. Emerging technologies are driving the development of sophisticated therapeutic platforms tailored to individual patient needs. By incorporating multi-omics data and precision medicine approaches, these platforms enable earlier diagnoses and personalized treatments (Rhodes et al., 2022). This convergence of diagnostic and therapeutic capabilities holds promise for providing more precise and effective solutions for PH patients.

The shift toward personalized and adaptive therapies is exemplified by recent advancements in biomaterials. For instance, RAGE-targeted peptide nanofibers have demonstrated significant accumulation in the lungs, enabling targeted and personalized treatments—a major step toward adaptive PH therapies (Marulanda et al., 2021). Additionally, programmable hydrogel systems have been developed to achieve dynamic drug release in response to environmental stimuli, demonstrating their potential in creating real-time adaptive therapeutic platforms for diseases like PH (Badeau et al., 2018). Integrating biomarker monitoring technologies with biomaterials makes therapeutic measures more precise in addressing disease progression. Tissue-informed biomaterials further advance this trend by supporting customized therapy based on individual patient attributes, significantly improving personalized PH therapies. In the future, combining diagnostic tools with therapeutic platforms is expected to provide real-time monitoring of PH pathology, further optimizing treatment outcomes.

Given the heterogeneity of PH subtypes, developing customized biomaterial platforms is critical for achieving optimal therapeutic outcomes. Modular scaffold systems can be designed to include patient-specific molecular cues, such as tailored growth factor profiles or hypoxia-responsive elements, to align with the unique pathophysiological features of different PH subtypes (Poh et al., 2022). The integration of omics-based diagnostic technologies enables patient stratification based on genomic, proteomic, and metabolomic signatures, guiding the personalized design of biomaterial constructs (Elinoff and Solomon, 2018). Precision-engineered biomaterials, developed through additive manufacturing approaches, hold significant promise for maximizing therapeutic efficacy while minimizing adverse effects, thus advancing the paradigm of individualized therapy for PH (Guzzi and Tibbitt, 2020).

To ensure the successful translation of biomaterials into effective PH treatments, continuous innovation is required in manufacturing technologies, regulatory compliance, and economic models. Biomaterial-based therapies, when combined with conventional treatment strategies, offer safer and more effective options while adhering to regulatory standards. The development of robust risk management frameworks ensures both safety and regulatory compliance, creating a solid foundation for personalized therapies in the future. One of the most pressing regulatory challenges in translating biomaterial-based therapies for PH into clinical practice is the ambiguity in their classification, which often blurs the boundaries between medical devices, biologics, and combination products. Regulatory agencies require comprehensive preclinical validation to address issues related to biocompatibility, immunogenicity, biodegradation, and long-term safety. In addition, stringent manufacturing controls to ensure batch-to-batch consistency, scalability, and reproducibility remain critical. Although new regulatory guidelines for regenerative biomaterials are emerging, standardized frameworks for multi-component nano–bio systems remain limited, posing significant barriers to the rapid clinical translation of these advanced therapies.

The potential of biomaterials in PH therapy extends far beyond current applications. Future therapeutic approaches will increasingly focus on tailoring treatments to individual patient needs while maintaining feasibility for widespread clinical adoption. Advances in biomaterials engineering continue to enhance drug delivery and precision medicine, establishing biomaterials as a cornerstone in PH treatment strategies. These advancements are poised to significantly improve long-term disease management and therapeutic outcomes for patients with PH (Aashish and Chettiar, 2024).

## 7 Conclusion

Biomaterials have emerged as pivotal tools for advancing PH therapy, offering new avenues for vascular regeneration, immune modulation, and precise localized drug delivery. Through dynamic responsiveness and integration with nanotechnology, biomaterial platforms are increasingly capable of adapting to the evolving pathophysiological landscape of PH.

The convergence of biomaterials with AI-driven design and multi-omics-guided personalized approaches further paves the way for next-generation customized interventions. However, successful clinical translation will require overcoming key challenges, including immunogenicity, long-term stability, manufacturing scalability, and regulatory hurdles. Future research should prioritize the development of adaptive, patient-specific biomaterials capable of dynamic remodeling and targeted intervention, ultimately aiming to shift PH management from symptomatic control toward true disease modification and vascular regeneration.
